# The Use of Mebendazole in COVID-19 Patients: An Observational Retrospective Single Center Study

**DOI:** 10.1155/2022/3014686

**Published:** 2022-12-10

**Authors:** Mostafa W. Galal, Mahmoud Ahmed, Yanqiu Shao, Chao Xing, Wael Ali, Abd Elhamid Baly, Abdallah Elfiky, Khaled Amer, John Schoggins, Hesham A. Sadek, Zeinab N. Gobara

**Affiliations:** ^1^School of Medicine, Aim Shams University, Cairo, Egypt; ^2^Department of Internal Medicine Cardiology, University of Texas Southwestern Medical Center, Dallas, TX 75390, USA; ^3^Department of Statistical Science, Southern Methodist University, Dallas, TX 75275, USA; ^4^Department of Population and Data Sciences, University of Texas Southwestern Medical Center, Dallas, TX 75390, USA; ^5^McDermott Center for Human Growth and Development and Department of Bioinformatics, University of Texas Southwestern Medical Center, Dallas, TX 75390, USA; ^6^Egyptian Center for Research in Regenerative Medicine, Cairo, Egypt; ^7^Department of Clinical Pathology, Cura El-Nasr Hospitals, Cairo, Egypt; ^8^Department of Radiology, Cura El-Nasr Hospitals, Helwan university, Cairo, Egypt; ^9^Department of Biochemistry, University of Texas Southwestern Medical Center, Dallas, TX 75390, USA; ^10^Epartments Microbiology, University of Texas Southwestern Medical Center, Dallas, TX 75390, USA; ^11^Departments Biophysics and Molecular Biology and Center for Regenerative Science and Medicine, University of Texas Southwestern Medical Center, Dallas, TX 75390, USA

## Abstract

**Background:**

An *in-silico* screen identified mebendazole with potential antiviral activity that could be a repurposed drug against SARS-CoV-2. Mebendazole is a well-tolerated and cheap antihelminthic agent that is readily available worldwide and thus could be a therapeutic tool in the fight against COVID-19.

**Methods:**

This is an observational retrospective study of PCR-confirmed COVID-19 patients who received mebendazole with the intention-to-treat. The study included an inpatient cohort (157 inpatients) and an outpatient cohort (185 outpatients). Of the 157 inpatients and 185 outpatients, 68 (43.3%) and 94 (50.8%) received mebendazole, respectively. Patients who presented within the same timeframe but did not receive mebendazole were used as controls. Patients received standard-of-care treatment including remdesivir, dexamethasone, and anticoagulants as deemed necessary by the treating physician. The following clinical outcomes were evaluated: for the inpatient cohort, length of stay (LOS) at the hospital, need for ventilation (combined invasive and noninvasive), and mortality; for the outpatient cohort, time to symptom resolution, need for hospitalization, and mortality.

**Results:**

For the inpatient cohort, the median age did not differ between the treatment and control groups; 62 (56, 67) vs. 62 (56, 68), *P*, and there was a comparable proportion of males in both groups; 43 (63%) vs. 55 (62%), *P*=0.85. The hospital LOS was 3.5 days shorter in the treatment group compared to the control group (*P* < 0.001). There were fewer patients who required invasive or noninvasive ventilation in the treatment group, 2 (2.9%) vs. 7 (7.9%), and the mortality rate is lower in the treatment group, 3 (4.4%) vs. 8 (9.0%), though the differences did not reach statistical significance. For the outpatient cohort, the median age was lower in the treatment group compared with the control group; 40 (34, 48) vs. 48 (41, 54), *P* < 0.001. There was a comparable proportion of males between both groups; 50 (53%) vs. 52 (57%), *P*=0.59. Patients in the treatment group were 3.3 days closer to symptom resolution (*P* < 0.001). There were numerically fewer patients requiring hospitalization in the treatment group compared with the control group, 3 (3.2%) vs. 6 (6.6%), though this did not reach statistical significance (*P*=0.33).

**Conclusion:**

In this retrospective observational study, the use of mebendazole in COVID-19 patients was associated with shorter hospitalizations in the inpatient cohort and shorter durations of symptom resolution in the outpatient cohort. The findings from this small observational study are hypothesis-generating and preclude drawing conclusions about clinical efficacy. Further studies are needed to examine the role of mebendazole in the treatment of COVID-19 patients.

## 1. Introduction

SARS-CoV-2 was identified in late December 2019 as the causative agent of a severe acute respiratory syndrome named COVID-19 [1–3]. COVID-19 was officially declared a pandemic by the World Health Organization (WHO) on March 11th, 2020. The virus has since propagated worldwide, causing an unprecedented incidence of morbidity and mortality. At the time this manuscript was written, there were 283 million reported cases and 5.41 million deaths worldwide. There are currently a large number of trials that are testing various antiviral agents that target viral proteins that are critical for viral replication. A clinicaltrials.gov search at the time this manuscript was written (*in August 2022*) found over *400* clinical trials testing antivirals against SARS-CoV-2 that are either active or pending, with over 100 similar clinical trials that have been completed. These antivirals include new and repurposed drugs targeting viral proteins that are critical for viral replication, such as the proteases (main protease (M^pro^) and papain-like protease) and RNA polymerase. For example, the first FDA-approved antiviral drug against SARS-CoV-2 was remdesivir, which was originally developed for the Ebola virus and has been successfully repurposed as a SARS-CoV-2 RNA polymerase inhibitor. Recently, Merck pharmaceuticals announced that molnupiravir, an oral antiviral agent, decreased the risk of hospitalization from COVID-19 by about 30% [4–6]. Also, Pfizer announced the clinical outcomes for their oral SARS-CoV-2 M^pro^ inhibitor, Paxlovid, that reduced the risk of hospitalization or death by 89% (within three days of symptom onset) and 88% (within five days of symptom onset) compared to placebo [7, 8]. Both drugs were granted by the U.S. FDA Emergency Use Authorization. These new drugs, however, are unlikely to be widely available worldwide soon.

In March 2020, a preprint identified a number of FDA-approved drugs with potential antiviral activity against SARS-CoV-2 in vitro [9]. This study performed an in-silico screen to identify FDA-approved drugs that can potentially inhibit SARS-CoV-2 M^pro^. One of the top hits identified in that report was mebendazole, which is a widely used benzimidazole broad-spectrum oral antihelminthic agent [10]. Subsequently, *in vitro* antiviral screening demonstrated the inhibitory profile of mebendazole on SARS-CoV-2 viral replication at low micromolar concentrations, which is achievable at therapeutic doses. The study also performed pharmacokinetic (PK) studies in mice, which confirmed drug levels in bronchoalveolar lavage (BAL) samples within the IC50 range. This is an important finding since the failure of many repurposed drugs is linked to the mismatch between IC50 concentrations and therapeutic drug levels that can be safely achieved in vivo. Mebendazole was first approved in 1971, and since then it has become widely available worldwide due to its effectiveness, cheap price, and low adverse effects; it is on the WHO list of essential medicines [11, 12]. Mechanistically, mebendazole is believed to inhibit microtubule formation in parasites, resulting in their eventual death [13, 14]. In addition, mebendazole is currently being studied as a potential anticancer agent, where it was found to impact a wide range of targets, including angiogenesis and multidrug resistance protein transporters, among others [15].

Given its availability, favorable side effect profile, and lack of widely available and cheap oral antiviral agents against SARS-CoV-2, mebendazole has been administered off-label to PCR-confirmed COVID-19 patients with an intention-to-treat. Here we report the results of a retrospective analysis of two patient cohorts from a single center in Cairo, Egypt.

## 2. Methods

### 2.1. Study Population

This is a single-center observational study that included all comers with a diagnosis of COVID-19 from June 1st to August 30, 2020. The diagnosis of COVID-19 pneumonia was confirmed in all patients via SARS-CoV-2 RT-PCR. Patients who required hospital admission (inpatients) and those who were managed at home (outpatients) were included. The decision to admit patients was based on the managing provider's discretion, guided by clinical conditions and laboratory parameters.

### 2.2. Management Protocol

All patients received standard care at the discretion of the treating providers. Treatment included remdesivir, prednisone, and anticoagulation. Patients were offered mebendazole as an alternate therapy to hydroxychloroquine and ivermectin (which were included in some COVID-19 treatment protocol guidelines in Egypt [16]) when these drugs were not available. Mebendazole was administered at 500 mg BID for a maximum duration of 14 days. The study population was then stratified into those who received mebendazole (treatment) and those who did not (control). The treatment assignment was not randomized and was based on the provider's discretion in line with the patient's preferences, and the patients were informed of this off-label treatment.

### 2.3. Statistical Analysis

Continuous variables are presented as the median (interquartile range; IQR), and discrete variables are presented as counts (percentage). Outcomes of interest for inpatients were mortality, hospital LOS, and the requirement of ventilation (either noninvasive such as CPAP and BiPAP or invasive mechanical ventilation). Outcomes of interest for outpatients were duration to symptom resolution (patient becoming afebrile in addition to symptomatic improvement of any of the following symptoms: cough, fatigue, and sore throat), hospital admission, and mortality. For demographic and clinical variables in Table 1, we compared the continuous variables using the two-sample *t*-test and the discrete variables using Fisher's exact test.

For discrete outcomes of interest variables in Table 2, Fisher's exact test was performed without adjusting for covariates due to the small number of events. In contrast, the effects of mebendazole treatment on the continuous variables—hospital LOS for the inpatients and duration of symptoms for the outpatients—were estimated by the fitting of multiple linear regression models including the demographic and clinical covariates backward selected by the Akaike information criterion. All statistical analyses were performed using *R* (v3.6.3).

## 3. Results

The study cohorts comprised 157 inpatients and 185 outpatients. Of the 157 inpatients and 185 outpatients, 68 (43.3%) and 94 (50.8%) received mebendazole, respectively. The comparisons of demographic and clinical variables are summarized in Table 1, and outcomes are summarized in Table 2.

### 3.1. Inpatient Cohort

#### 3.1.1. Baseline characteristics

The median age (year) did not differ between the treatment and the control groups, 62 (56, 68) vs. 62 (56, 67), and there were almost equal proportions of males in both groups, 43 (63.2%) vs. 55 (61.8%). The prevalence of diabetes and hypertension was low and balanced between the two groups.

#### 3.1.2. Vital signs on Admission

The baseline heart rate (HR) and the respiratory rate (RR) did not differ between the two groups. However, the median temperature (degrees Celsius) was lower in the treatment group, 38.6 (38.2, 39.2) vs. 39.1 (38.5, 39.5), *P*=0.0012, and the oxygen saturation (sO2, %) was lower in the treatment group, 85% (83.5%, 87.5%) vs. 90% (89%, 92%), *P* < 0.001.

#### 3.1.3. Lab values on Admission

Baseline lab values, including ferritin, D-dimer, and lactate dehydrogenase (LDH) did not differ between the two groups. The median lymphocyte count (per mL) was slightly higher in the treatment group, 1.1 (0.8, 1.5) vs. 0.9 (0.7, 1.2), *P*=0.041, and the median CRP level (mg/dL) was lower in the treatment group, 48 (36, 95) vs. 64 (49, 82), *P*=0.013. There was a higher percentage of patients with abnormal kidney function tests (RFTs) in the treatment group, 33.8% vs. 16.9%, *P*=0.016. However, there was no difference in the percentage of patients with abnormal liver function tests (LFTs).

#### 3.1.4. Medications received

All patients received anticoagulation. But 5 patients in the treatment group received steroids, 63 (92.6%) vs. 89 (100%), *P*=0.014. Slightly fewer patients in the treatment group received remdesivir, 35 (51.5%) vs. 58 (65.2%), *P*=0.1.

#### 3.1.5. Outcomes

There were fewer patients who required either invasive or noninvasive ventilation in the treatment group, 2 (2.9%) vs. 7 (7.9%), and the mortality rate is lower in the treatment group, 3 (4.4%) vs. 8 (9.0%), though the differences did not reach statistical significance. However, excluding those deceased, the hospital LOS (day) was significantly shorter in the treatment group, 7 (6, 9) vs. 10 (9, 12), *P* < 0.001. Adjusting for the demographic and clinical covariates, the hospital LOS of patients in the treatment group was 3.5 days shorter than those in the control group (*P* < 0.001).

### 3.2. Outpatient Cohort

#### 3.2.1. Baseline characteristics

The median age (year) was lower in the treatment group compared with the control group, 40 (34, 48) vs. 48 (41, 54), *P* < 0.001. There was a comparable proportion of males between both groups; 50 (53.2%) vs. 52 (57.1%).

#### 3.2.2. Vital signs on Admission

Baseline temperature (Celsius), HR (beats/min), and RR (breaths/min) were statistically different but clinically similar between the treatment and control groups— 38.5 (38.1, 38.9) vs. 38.8 (38.3, 39.4), *P*=0.0011; 97 (92, 100) vs. 94 (86, 100), *P*=0.0052; and 20 (18, 21) vs. 19 (17, 20), *P* < 0.001, respectively. There was no documented hypoxia in either group, 94 (93, 95) vs. 94 (93, 95).

#### 3.2.3. Lab values on Admission

Lymphocyte count and D-dimer did not differ between the groups. The median ferritin (ng/ml), CRP, and LDH levels were lower in the treatment group compared to the control group— 440 (361, 553) vs. 522 (418, 650), *P* < 0.001; 25 (18, 36) vs. 37 (27, 48), *P* < 0.001; 473 (362, 561) vs. 542 (418, 653), *P*=0.0013. There were almost equal numbers of patients in the treatment and control groups with abnormal RFTs and LFTs—6 (6.4%) vs. 6 (6.6%) and 7 (7.4%) vs. 6 (6.6%), respectively.

#### 3.2.4. Medications received

Fewer patients in the treatment group received steroids, 8 (8.5%) vs. 20 (22.0%), *P* < 0.001, but a similar proportion received anticoagulation, 10 (10.6%) vs. 9 (9.9%). None of the patients in either group received remdesivir.

#### 3.2.5. Outcomes

None of the patients were deceased. There were numerically fewer patients requiring hospitalization in the treatment group compared to the control group, 3 (3.2%) vs. 6 (6.6%), though this did not reach statistical significance. However, excluding those transferred to hospitals, the patients in the treatment group had a shorter duration of symptoms resolution (day), 4 (3, 4) vs. 7 (6, 8), *P* < 0.001. Adjusting for the demographic and clinical covariates, the time to symptom resolution of patients in the treatment group was 3.3 days shorter than those in the control group *P* < 0.001.

## 4. Discussion

The rapid devastation caused by COVID-19 has prompted an unprecedented race to find safe and effective therapies to reduce the morbidity and mortality caused by this once-in-a-century pandemic. Not surprisingly, repurposing approved drugs has been at the forefront of these efforts, as this allows prioritization of drug candidates with known favorable safety profiles. Hundreds of basic and clinical studies identify potential disease modifiers, ranging from immune modulators to drugs targeting various host and viral pathways involved in viral entry or replication. In the current retrospective study, an association was observed between mebendazole use and improved clinical outcomes in both inpatients and outpatients.

No increase was observed in mortality, worsening clinical outcomes, or abnormal laboratory findings associated with mebendazole treatment in the current study. In addition, no patients discontinued mebendazole due to intolerance. It is important to note here that the maximal dose of mebendazole typically used for hydatid disease is up to 50 mg/kg/day for up to 4 weeks [17], and thus the dose used in the current study is considered modest (all patients weighed >50 kgs). The dose of 500 mg BID used in the current study was primarily driven by the availability of 500 mg pills in Egypt (*trade name Verm1*).

From a pharmacokinetic perspective, the reported IC50 for mebendazole on SARS-CoV-2 in Vero6 cells was approximately 1.5 *μ*M. Mebendazole is highly plasma protein-bound and has a volume of distribution of 1–2 L/Kg, indicating that it is not restricted to the intravascular compartment. Rodent PK studies reported earlier indicate that free mebendazole concentrations in BAL samples exceed the IC50 observed in vivo, which suggests that, from a theoretical perspective, it is possible for therapeutic doses of mebendazole to be achieved in COVID-19 patients, specifically in the lung tissue. It is important to note, however, that we did not perform PK studies in humans and we are unsure of the in vivo IC50 needed for inhibition of viral replication, and thus these observations, although based on in vitro and animal data, remain speculative from a clinical perspective.

Mebendazole is generally well tolerated, with mild reported adverse effects including abdominal pain, diarrhea, nausea, flatulence, vomiting, and loss of appetite. Few patients had experienced severe and rare adverse effects, such as seizures, convulsions, and hypersensitivity reactions [18]. Mebendazole toxicity is mainly related to gastrointestinal adverse effects, but higher doses can lead to neutropenia and thrombocytopenia, which have direct contraindications for COVID-19 patients [19]. Since the administered regimen is within the therapeutic index of mebendazole, we did not observe any significant side effects that required the drug to be discontinued in the current population. It is important to note that there were some differences in patient demographics and baseline characteristics that may have impacted the observed results. For example, for the inpatient cohort, the median temperature and oxygen saturation on admission were slightly lower in the mebendazole group. In addition, the mebendazole group had lower CRP levels and a higher percentage of patients with abnormal renal function. Importantly, fewer patients in the mebendazole group received corticosteroids. For the outpatient cohort, pertinent differences include the median age, which was 8 years lower in the mebendazole group. It is unclear how these patient demographics may have impacted the overall observed effect. For example, while younger age is associated with better prognosis and lower rates of complications [20], lower oxygen saturation [21] and abnormal renal function [22] upon presentation are associated with worse prognosis.

As noted earlier, numerous approved drugs have been tested in COVID-19 patients following initial observations of antiviral effects in tissue culture. Notable examples include hydroxychloroquine and ivermectin [23, 24], both of which have been used worldwide and even implemented in treatment protocols. An important caveat of using these two drugs, despite their clear antiviral effect in vitro, is the unfavorable pharmacokinetic profiles. Both of them showed an IC50 against SARS-CoV-2 in the low micromolar range, which is not achievable to meet the therapeutic plasma concentrations in vivo. Eventually, higher doses of ivermectin are required to attain the therapeutic plasma concentrations, which can lead to serious adverse effects such as ataxia, convulsions, severe neurotoxicity, and coma [25]. In contrast, we have previously performed PK studies on mebendazole in mice, which indicate that the therapeutic plasma concentration for the administered dosage regimen in vivo is well correlated with the reported IC50 ranges against SARS-CoV-2 in vitro [26].

Finally, it is imperative to caution against using data from the current observational study to guide therapy. Results from the current study should be considered hypothesis-generating to guide decision-making for potential future randomized controlled clinical trials. We conclude that mebendazole administered at 500 mg BID appears to be safe in COVID-19 patients in both inpatient and outpatient settings, and that future randomized controlled clinical trials are warranted to evaluate the efficacy of mebendazole as an oral antiviral agent against SARS-CoV-2.

## Figures and Tables

**Table 1:** Demographics and clinical variables of the mebendazole treatment group and nonmebendazole treatment group for both inpatients and outpatients.

	Inpatients			Outpatients		
	Control	Treatment	*P* value	Control	Treatment	*P* value
N	89	68		91	94	
Demographics						
Age, year	62 (57, 67)	62 (56, 68)	0.55	48 (41, 54)	40 (34, 48)	2.4 × 10^−6^
Sex, male	55 (61.8)	43 (63.2)	0.87	52 (57.1)	50 (53.2)	0.66
Comorbidity						
Diabetes mellitus	7 (7.9)	6 (8.8)	1	4 (4.4)	5 (5.3)	1
Hypertension	7 (7.9)	9 (13.2)	0.30	2 (2.2)	2 (2.1)	1

Vital signs on admission						
Temperature (Celsius)	39.1 (38.5, 39.5)	38.6 (38.2, 39.2)	1.2 × 10^−3^	38.8 (38.3, 39.4)	38.5 (38.1, 38.9)	1.1 × 10^−3^
Heart rate	100 (98, 105)	100 (95, 105)	0.65	94 (86, 100)	97 (92, 100)	5.2 × 10^−3^
Respiratory rate	20 (19, 22)	21 (19, 22)	0.61	19 (17, 20)	20 (18, 21)	2.3 × 10^−4^
sO2	90 (89, 92)	85 (83.5, 87.5)	1.5 × 10^−21^	94 (93, 95)	94 (93, 95)	0.85

Lab values						
Lymphocyte count	0.9 (0.7, 1.2)	1.1 (0.8, 1.5)	4.1 × 10^−2^	0.9 (0.7, 1.1)	0.8 (0.6, 0.9)	6.5 × 10^−2^
Ferritin	680 (361, 897)	592 (387, 817)	0.63	522 (418, 650)	440 (361, 553)	1.6 × 10^−5^
CRP	64 (49, 82)	48 (36, 95)	1.3 × 10^−2^	37 (27, 48)	25 (18, 36)	1.2 × 10^−7^
D-dimer	1.1 (0.8, 1.8)	1.14 (0.81, 1.71)	0.49	0.9 (0.7, 1.1)	0.8 (0.7, 1.1)	0.60
LDH	648 (487, 754)	669 (527, 780)	0.35	542 (418, 653)	473 (362, 561)	1.3 × 10^−3^
Abnormal LFT	16 (18.0)	14 (20.6)	0.69	6 (6.6)	7 (7.4)	1
Abnormal RFT	15 (16.9)	23 (33.8)	1.6 × 10^−2^	6 (6.6)	6 (6.4)	1

Medications received						
Remdesivir	58 (65.2)	35 (51.5)	0.10	0 (0)	0 (0)	1
Steroids	89 (100)	63 (92.6)	1.4 × 10^−2^	20 (22.0)	8 (8.5)	1.3 × 10^−2^
Anticoagulation	89 (100)	68 (100)	1	9 (9.9)	10 (10.6)	1

Continuous variables are presented as medians (interquartile range) and discrete variables are presented as counts (percentage). Continuous variables were compared using the two-sample *t*-test, and discrete variables were compared using Fisher's exact test. CRP: C-reactive protein; LFT: liver function tests; RFT: renal function tests.

**Table 2:** Outcomes of the mebendazole treatment group and the nonmebendazole treatment group for both inpatients and outpatients.

	Inpatients			Outpatients		
	Control	Treatment	*P* value	Control	Treatment	*P* value
N	89	68		91	94	
Death	8 (9.0)	3 (4.4)	0.35	0 (0)	0 (0)	1
Ventilation	7 (7.9)	2 (2.9)	0.30	—	—	—
Hospital LOS	10 (9, 12)	7 (6, 9)	6.0 × 10^−8^	—	—	—
Need for hospitalization	—	—	—	6 (6.6)	3 (3.2)	0.33
Time to symptom resolution	—	—	—	7 (6, 8)	4 (3, 4)	6.6 × 10^−42^

Continuous variables are presented as medians (interquartile range) and discrete variables are presented as counts (percentage). Continuous variables were compared by fitting multiple linear regression models, including the demographic and clinical covariates backward selected by the Akaike information criterion; discrete variables were compared using Fisher's exact test.

## Data Availability

The data will be made available upon request in accordance with institutional policy.
